# COVID en Español: Reflections of a trauma therapist serving Spanish-speaking Latinx survivors of violence

**DOI:** 10.1177/1473325020973324

**Published:** 2021-03

**Authors:** Yaneth Lombana

**Affiliations:** The Graduate Center CUNY, New York, USA

**Keywords:** Latino, trauma, therapy, critical reflection, cultural competence, patients’ experiences

## Abstract

In this reflexive essay I share my experiences as a trauma-focused psychotherapist serving Spanish-speaking Latinx survivors of violence in New York City during the COVID-19 pandemic. Successes and challenges of working with this population during the pandemic are highlighted and connected to broader realities in the mental health field. Vicarious trauma is presented from the lens of a practitioner who shares a similar background to the population served.

In this reflexive essay I share my experiences as a trauma-focused psychotherapist serving Spanish-speaking Latinx survivors of violence in New York City during the COVID-19 pandemic. Successes and challenges of working with this population during the pandemic are highlighted and connected to broader realities in the mental health field. Vicarious trauma is presented from the lens of a practitioner who shares a similar background to the population served.

When I asked her, “¿Estás en un lugar dónde podemos hablar en privado por los próximos 45 minutos?” (Are you in a place where you can speak in private for the next 45 minutes?), she answered, “Sí, estoy en las escaleras” (Yes, I’m in the stairwell). “¿No quieres tomar la llamada en tu apartamento?” (You don’t want to take the call in your apartment?), I asked. “No,” she said, “hay demasiados recuerdos ahí” (There are too many memories there). As a trauma therapist working with Spanish-speaking crime survivors, I often find myself adjusting methodologies–most often created by and for Europeans and Americans–to best fit my clients. However, starting sessions with this one question is very much a product of our times.

Most of my clients are Latinx Spanish speakers even though for some, Spanish (our colonizers’ language) is not their primary tongue. Latinx trauma survivors’ realities coupled with the mental health field’s abysmally low numbers of clinicians of color (Lin et al., 2018) create multiple barriers to treatment. Prior to the COVID-19 pandemic, some of my clients would commute long distances to have a session with the one Spanish-speaking trauma-focused Latinx therapist available at our organization: me. Additionally, parents of my child clients must navigate their work schedule, school pick-ups, and, at times, childcare to attend our weekly sessions because the treatment modality we use requires active caregiver participation.

Having an already established relationship with my clients made our “pandemic adjustments” less tricky to manage. Technical changes such as new forms of communication were a matter of discussion and trial. Children transitioned to online meetings, since phone interactions were less conducive to engagement and they were already used to communicating in this way for school. Adults switched to phone calls because they expressed this to be their preference. For some parents, switching to a technological platform brought new stressors. At least one of my clients cannot read but even the ones who are quite tech savvy preferred not associating their therapy with another online meeting.

To my surprise, these technical changes, rather than acting as barriers, made it easier for my clients to be consistently engaged in treatment. No longer were commutes and childcare an impediment for participation. Additionally, because we had been working together for at least a couple of months, we were connected enough that even through the phone we could reach the important breakthrough moments that we had only experienced in person. Usually these moments are important in moving therapy forward; however, now, in a time when people feel isolated, fears are heightened, and daily difficulties have increased, these therapeutic moments can feel even more valuable and harder to achieve.

I remember during a processing session telling one client, “Puedes llorar” (It’s okay to cry) and hearing her as she let her tears flow. This was the same client who told me: “Cuando hablamos, te imagino sentada frente a mí, detesto no poder verte” (When we speak, I picture you sitting in front of me, I hate not seeing you). I was amazed at the fact that even through a phone, I could connect with her tone, envision her body language, and read the moment with the exactitude I can in person. I knew that my ability to read and connect with my clients’ feelings had to do with the way mirror neurons work (Berrol, 2006, Lamm et al., 2007), but thought the lack of physical proximity would interfere with the precision with which I can normally be in tune with my clients.

In spite of these victories, providing psychotherapy during a global pandemic has added layers of barriers to an already delicate endeavor. The clients I work with have complex trauma, regularly experience discrimination, and often navigate systems that do not treat them with dignity. The pandemic–through exposure to news coverage or via lived experience–brought to the surface past dire situations at the same time that it added new ones. For one undocumented client who had experienced human trafficking and is currently out of a job, images and information about potential food shortages ignited memories of times they experienced extreme poverty. As a result, their reactions were more intense than those of an average person who experienced fear elicited by food insecurity. For a domestic violence survivor with multiple children in a small New York City apartment, being in quarantine required creativity to keep her children active and well (something that’s already difficult to achieve under normal circumstances) while also learning to manage her children’s and her own trauma reactions with limited coping options. For those who lost loved ones, the pain of death was intensified by not being able to have a traditional service: something that helps provide closure and a space for rituals valued by the family and the collective centric community.

Trauma impacts not only the person who lived it directly but also those in their proximity. The people we commune with are implicated in the types of conversations we have, how we react to things that remind us of what we lived, and the modifications we need to reach a feeling of safety and comfort. The nature of the work I do means that I too am impacted by it (Van Dernoot Lipsky and Burk, 2009). I connect with the pain of the individual but because I am working with my community, I also connect with this pain at multiple levels. These effects, known as vicarious trauma, can show up so subtly that it may be difficult to notice. In spite of my knowledge of how it works, awareness of when I’m experiencing vicarious trauma is not as evident. For example, about a year and a half into my current job, I showed up at the Department of Motor Vehicles with so much documentation that I was able to obtain an enhanced license: a document that allows you to cross the US border without a passport. Days later I realized I hadn’t brought all my documentation because I wanted an enhanced license–I had renewed my license enough times to know I just needed to bring my old one–but that I connected my presence in a government building where I had to “show papers” with the fear my undocumented clients experience. Then I was able to put the pieces together: the tightness in my body as I spoke to the first person at the door, my inability to concentrate once inside, and the additional documentation I had brought with me, were all signs that I was embodying (Van Der Kok, 2014) the client experiences I hold.

It’s my job to support people in identifying how to best manage their reactions to things that connect them to extreme experiences they lived, to help them realize how these experiences have impacted them and their relationships, and to process their experiences to shift to a life with a panoramic scope that holds a view of new possibilities that reflect mastery over the lived traumatic experiences. At the same time, for my well-being, and to do my job in the best way possible, I must also be aware of how my clients’ trauma impacts me: something that has become even more elusive during a time where I, as a Latinx and New York resident, am too experiencing threats to my livelihood and a decrease in access to resources for coping.

My clients who recently completed treatment taught me that with the right support, moving beyond trauma is possible even within a globally occurring pandemic. It may be awhile before I am fully aware of the effects of bearing witness to the processes lived by my community as I work with them to reach for a better state during the COVID-19 pandemic. It is not a smooth journey, but it is one that continues to fill me with pride: to be providing culturally competent services and learning opportunities as I further extend my expertise in trauma. In this process, I am seeing my community heal and it is an absolutely beautiful and overwhelmingly joyous thing. I just wish I had more fellow therapists to share this with en español.
